# Association of neutrophil-lymphocyte ratio with all-cause and cardiovascular mortality in US adults with diabetes and prediabetes: a prospective cohort study

**DOI:** 10.1186/s12902-024-01592-7

**Published:** 2024-05-10

**Authors:** Guangshu Chen, Li Che, Meizheng Lai, Ting Wei, Chuping Chen, Ping Zhu, Jianmin Ran

**Affiliations:** 1 Department of Endocrinology and Metabolism, Guangzhou Red Cross Hospital, Jinan University, Guangzhou, 510220 Guangdong China; 2 State Key Laboratory of Respiratory Disease, National Clinical Research Center for Respiratory Disease, Guangzhou Institute of Respiratory Health, The First Affiliated Hospital of Guangzhou Medical University, Guangzhou, 510120 China; 3 Department of Pulmonary and Critical Care Medicine, The First Affiliated Hospital of Jinan University, Guangzhou, 510630 China; 4 Department of Hematology, Guangzhou Red Cross Hospital, Jinan University, Guangzhou, 510220 China

**Keywords:** Diabetes, Prediabetes, Neutrophil-lymphocyte ratio, All-cause mortality, Cardiovascular mortality, Cohort study

## Abstract

**Background:**

The neutrophil-lymphocyte ratio (NLR) is a novel hematological parameter to assess systemic inflammation. Prior investigations have indicated that an increased NLR may serve as a potential marker for pathological states such as cancer and atherosclerosis. However, there exists a dearth of research investigating the correlation between NLR levels and mortality in individuals with diabetes and prediabetes. Consequently, this study aims to examine the connection between NLR and all-cause as well as cardiovascular mortality in the population of the United States (US) with hyperglycemia status.

**Methods:**

Data were collected from a total of 20,270 eligible individuals enrolled for analysis, spanning ten cycles of the National Health and Nutrition Examination Survey (NHANES) from 1999 to 2018. The subjects were categorized into three groups based on tertiles of NLR levels. The association of NLR with both all-cause and cardiovascular mortality was evaluated using Kaplan-Meier curves and Cox proportional hazards regression models. Restricted cubic splines were used to visualize the nonlinear relationship between NLR levels and all-cause and cardiovascular mortality in subjects with diabetes after accounting for all relevant factors.

**Results:**

Over a median follow-up period of 8.6 years, a total of 1909 subjects with diabetes died, with 671 deaths attributed to cardiovascular disease (CVD). And over a period of 8.46 years, 1974 subjects with prediabetes died, with 616 cases due to CVD. The multivariable-adjusted hazard ratios (HRs) comparing high to low tertile of NLR in diabetes subjects were found to be 1.37 (95% CI, 1.19–1.58) for all-cause mortality and 1.63 (95% CI, 1.29–2.05) for CVD mortality. And the correlation between high to low NLR tertile and heightened susceptibility to mortality from any cause (HR, 1.21; 95% CI, 1.03–1.43) and CVD mortality (HR, 1.49; 95% CI, 1.08–2.04) remained statistically significant (both *p*-values for trend < 0.05) in prediabetes subjects. The 10-year cumulative survival probability was determined to be 70.34%, 84.65% for all-cause events, and 86.21%, 94.54% for cardiovascular events in top NLR tertile of diabetes and prediabetes individuals, respectively. Furthermore, each incremental unit in the absolute value of NLR was associated with a 16%, 12% increase in all-cause mortality and a 25%, 24% increase in cardiovascular mortality among diabetes and prediabetes individuals, respectively.

**Conclusions:**

The findings of this prospective cohort study conducted in the US indicate a positive association of elevated NLR levels with heightened risks of overall and cardiovascular mortality among adults with diabetes and prediabetes. However, potential confounding factors for NLR and the challenge of monitoring NLR’s fluctuations over time should be further focused.

**Supplementary Information:**

The online version contains supplementary material available at 10.1186/s12902-024-01592-7.

## Background

The increasing rates of diabetes worldwide and the high number of diabetes-related deaths, especially from cardiovascular issues, have led to a focus on identifying factors that can predict mortality in individuals with diabetes [1–3]. Extensive research has consistently revealed a strong association between cardiovascular disease and inflammatory biomarkers [4, 5]. The body’s innate (neutrophils) and adaptive (lymphocytes) immune responses are balanced by the neutrophil-to-lymphocyte ratio [6]. It has recently gained recognition as a valuable indicator of systemic inflammation, encompassing both infectious and non-infectious conditions, such as cardiovascular disease [7, 8], tumors [9–12], septicemia [13, 14], and mental disorders [15, 16]. The development of diabetes may be caused by chronic inflammation, according to research [17, 18].

However, because of the related costs and measurement challenges, the use of several inflammatory markers in ordinary clinical practice has been restricted. Neutrophils’ negative effects on blood vessel linings are measured with NLR, an easy-to-use, affordable test based on well-studied white blood cell traits. Few cohort studies have examined the relationship between NLR levels and long-term health effects [19–22]. Regrettably, most of these studies have mainly concentrated on investigating the correlation between NLR and diabetes-related complications. To date, there has been limited scholarly investigation into the correlation between NLR and mortality among individuals with diabetes and prediabetes. Thus, the goal of this study is to look at the relationship between NLR and cardiovascular and overall mortality in US adults with hyperglycemia.

## Methods

### The design and population of the study

**Fig. 1:**
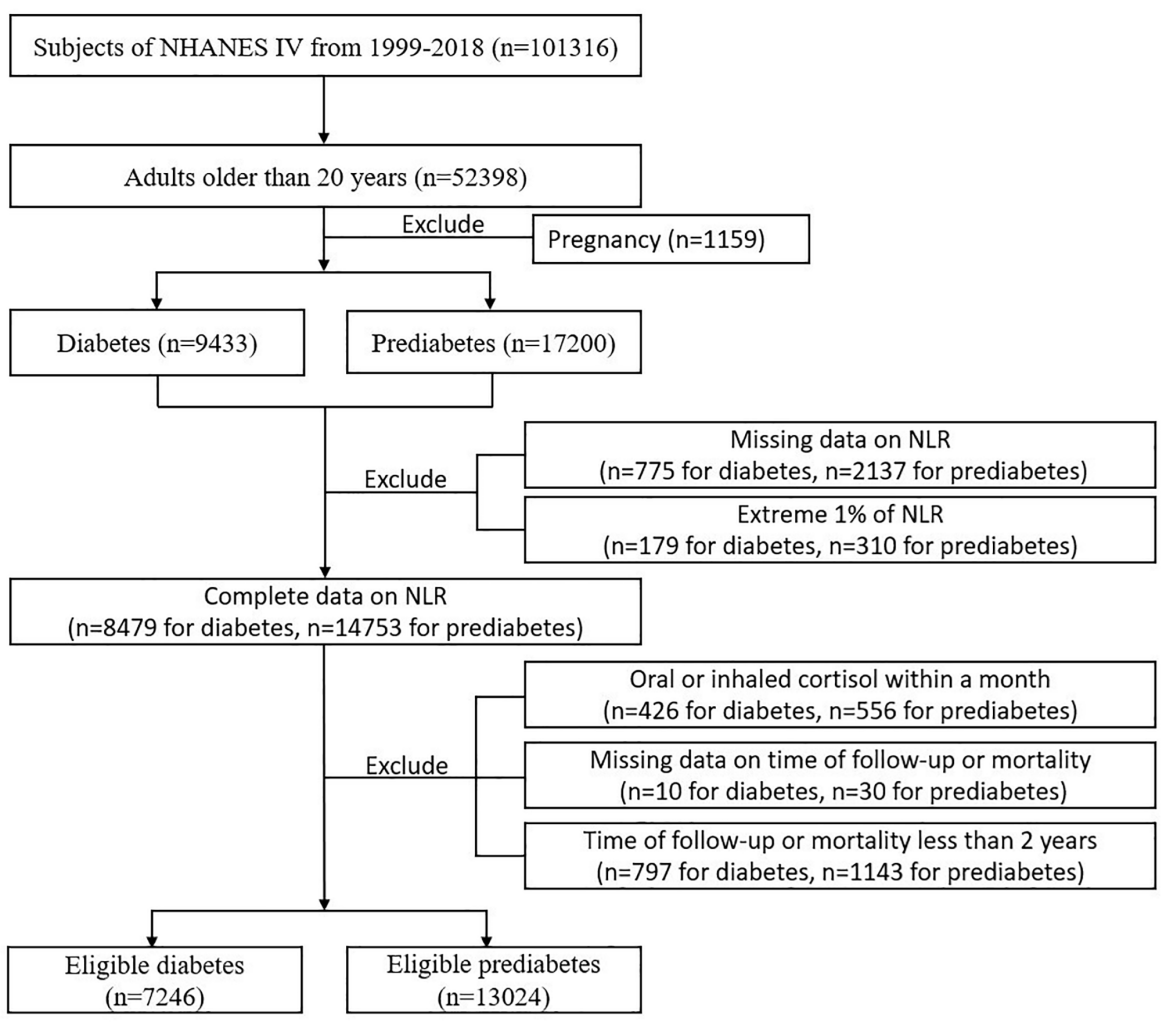
Flowchart about the inclusion and exclusion of eligible subjects

A population-based cross-sectional survey, the NHANES was created expressly to collect detailed information on the health and nutritional status of US households. The NHANES interview component encompasses inquiries on demographics, socioeconomic factors, dietary habits, and health-related matters and is accessible to external researchers. The NHANES study methodology has been extensively described by the US Centers for Disease Control and Prevention [23]. The National Center for Health Statistics granted approval for NHANES, and each participant gave written consent. Data from 10 cycles of NHANES conducted between 1999 and 2018 were utilized. Initially, a total of 52,398 individuals aged 20 years and above were included. Subsequently, 1159 cases of pregnant women were excluded, screening out 9433 diabetes cases and 17,200 prediabetes cases according to diagnosis standards. Then we exclude cases without complete data on NLR and within 1% extreme of NLR. To consider the possible influence of glucocorticoid-steroid usage on neutrophil and lymphocyte levels, extra individuals who had consumed oral or inhaled cortisol in the previous month were not included. Furthermore, cases were excluded due to factors such as inadequate follow-up time, and mortality within two years. In the analysis, a grand total of 7246 cases of eligible diabetes and 13,024 cases of eligible prediabetes were ultimately considered (refer to Fig. 1).

Based on NLR tertiles, baseline characteristics of participants with diabetes and prediabetes were acquired. The study employed weighted Kaplan-Meier (KM) survival curves to investigate differences in overall and CVD mortality among different NLR levels. Cumulative survival rates were presented in a risk table as weighted percentages. Among hyperglycemia subjects, dose-response relationships between NLR and mortality were demonstrated using restricted cubic splines (RCS) curves. The RCS curves depicted hazard ratios and 95% confidence intervals (CIs) through a solid line and gray shading. Any variants that influence neutrophil and lymphocyte counts were considered in our models to adjust the association between NLR and mortality. The models were modified to account for factors such as age, gender, ethnicity, level of education, ratio of family income to poverty, drinking habits, smoking habits, BMI, eGFR, HbA1c levels, duration of diabetes, medication for lowering glucose, CVD, hypertension, hyperlipidemia, cancer, chronic obstructive pulmonary disease (COPD), depression, and anemia. We further stratified different confounders to see the interaction effect on the association of NLR with overall and CVD mortality.

### Assessment of hyperglycemia and NLR

Diabetes was characterized by fulfilling any of the subsequent conditions: surpassing 7.0 mmol/L in fasting plasma glucose levels, having random plasma glucose levels or 2 h-glucose of 75-g oral glucose tolerance above 11.0 mmol/L, exhibiting HbA1c levels of 6.5% or greater (with serum hemoglobin level higher than 100 g/dL), utilizing insulin or self-reporting a medical professional’s diagnosis. Prediabetes was diagnosed according to one of the following conditions: fasting plasma glucose levels being 5.6-7.0mmol/L, random plasma glucose levels or 2 h-glucose of 75-g oral glucose tolerance being 7.8–11.0 mmol/L, HbA1c levels being 5.7–6.4% (with serum hemoglobin level higher than 100 g/dL), or self-reported history.

Automated hematology analyzing devices were used to obtain the counts of lymphocytes and neutrophils, with the unit expressed as ×1,000 cells/mm3. To calculate the neutrophil-to-lymphocyte ratio, divide the count of neutrophils by the count of lymphocytes. We categorized NLR into tertiles to further explore the relationship between different levels of NLR and mortality.

### All-cause and CVD mortality ascertainment

Mortality data, including all-cause and cardiovascular disease outcomes, were obtained from the National Death Index linked to the NHANES database until the end of December 2019.

The follow-up period persisted until the time of death or the conclusion of the period, starting from the date of blood analysis. The International Classification of Diseases, Tenth Revision (ICD-10) codes I00–09, I11, I13, I20–51, and I60–69 were used to identify mortality related to cardiovascular disease.

### Covariates assessment

Baseline data on eligible respondents were gathered using the Computer-Assisted Personal Interviewing (CAPI) system and the Family and Sample Person Demographics questionnaires. This data included information on age, gender, ethnicity, education level, family income-poverty ratio, smoking and drinking habits, usage of glucose-lowering medication, healthy eating index (HEI) scores, and past medical history such as cardiovascular disease (coronary heart disease, congestive heart failure, angina, heart attack or stroke), hypertension, cancer, and COPD. The NHANES protocol was used to assign weights to all baseline data. Using the physical examination data from NHANES, the body mass index (BMI) was calculated by dividing the body weight (measured in kilograms) by the square of the height (measured in meters). The study collected neutrophil, lymphocyte, and hemoglobin counts from a peripheral whole-blood test. Furthermore, serum creatinine, HbA1c, TC, LDL-C, HDL-C, TG, and fasting glucose were obtained through laboratory tests. To guarantee precise and uniform blood test procedures, the NHANES followed the Laboratory Procedure Manual. Interviewers recorded the duration of diabetes, considering newly diagnosed cases as having a duration of 0 year. The CKD-EPI equation was utilized to determine the estimated glomerular filtration rate (eGFR). Hyperlipidemia was characterized by fulfilling any of the subsequent conditions: total cholesterol (TC) levels equal to or exceeding 200 mg/dL, triglyceride (TG) levels equal to or surpassing 150 mg/dL, high-density lipoprotein cholesterol (HDL-C) levels less than or equal to 40 mg/dL in males and 50 mg/dL in females, low-density lipoprotein cholesterol (LDL-C) levels equal to or exceeding 130 mg/dL, or self-reported utilization of medications for reducing cholesterol. The depression group was identified using the PHQ-9 [24]. Depression status was defined as having a depression score greater than four. Anemia was diagnosed by establishing the serum hemoglobin (Hb) threshold (g/dL) for different demographic groups (non-pregnant women over 15 years old with levels below 120 g/dL, and men over 15 years old with levels below 130 g/dL). The HEI-2015 scores were employed as an indicator of dietary quality, with a higher score indicating a more nutritionally balanced diet [25]. MET scores were utilized to evaluate physical activity levels following the national physical activity guidelines (low physical activity being defined as less than 500 MET/wk and high physical activity being defined as 500 MET/wk or more) [26].

### Statistical analysis

Cox proportional hazards regression after survey-weight was used to explore the association of NLR level with overall and CVD-specific mortality based on different models in diabetes and prediabetes groups. All data were adjusted for survey weights in accordance with the analytic guidelines provided by NHANES due to the complex design. Whereas continuous data were shown as mean (standard error), categorical variables were shown as numbers (percentages). To investigate the disparities among groups in terms of baseline characteristics, we employed Weighted Chi-Square tests and Kruskal-Wallis tests. Using several models in the diabetic and prediabetes groups, Cox proportional hazards regression was used to assess the internal connection between NLR levels and overall and cardiovascular-specific death. The hazard ratios and 95% CIs were derived through survey-weighted calculations. We conducted three models to explore the relationship. Model 1 took into account factors such as age (below 65 or over 65), sex (male or female), race/ethnicity (Hispanic Mexican, non-Hispanic Black, non-Hispanic White, or others), marital status (married/cohabiting, single), family income-to-poverty ratio (< 1.0, 1.0–3.0, or ≥ 3.0), and education level (less than high school, high school or equivalent, or college or above). Model 2 included BMI (< 30 or ≥ 30.0 kg/m2), smoking status (never, former, or current), drinking status (non-drinker or ever drinker), physical activity (low or high), HEI scores, and cancer (no or yes) as additional adjustments. Model 3 further adjusted for eGFR (< 30, 30–60, >=60 ml/min/1.73 m²), anemia (no or yes), hypertension (no or yes), hyperlipidemia (no or yes), depression (no or yes), COPD (no or yes), use of hypotensive drug (no or yes), and use of lipid-lowering drug (no or yes) based on model 2. Diabetes duration, HbA1c levels, and use of antidiabetic drugs were adjusted additionally for diabetes subjects on model 3. The study employed statistical analysis to establish the first tertile of NLR as the reference group for evaluating the correlation between moderate-high NLR levels and mortality across various models. To handle missing values in the covariates of the study, we employed the technique of multiple imputation [27].

Weighted KM curves were employed to depict cumulative overall and CVD survival probability, stratified by tertiles of NLR levels. The risk table presented precise information regarding deaths and survival probability at different follow-up intervals. After fully adjusting for the mentioned covariates, RCS curves were utilized to visually depict the nonlinear correlation between NLR levels and both overall and CVD mortality in individuals with diabetes and prediabetes.

Subgroup analyses were performed to look at the relationship between NLR levels and death in people with diabetes and prediabetes. The subgroups were categorized using a variety of clinical and demographic characteristics, including age, gender, ethnicity, education, BMI, drinking and smoking habits, physical activity levels, and the occurrence of cancer, hypertension, and CVD. To ensure the strength of our findings, we performed sensitivity analyses. A particular analysis was carried out on individuals who had a prior record of cardiovascular disease or not. Following that, individuals who reported having no CVD and cancer at the baseline condition were also analyzed. The data underwent analysis using R software version 4.2.2 (R Foundation for Statistical Computing, Vienna, Austria), and a two-sided *P*-value < 0.05 was utilized to ascertain statistical significance.

## Results

### Baseline characteristics analyses

Our study included a total of 7246 and 13,024 adults aged 20 years or older who had been diagnosed with diabetes and prediabetes, respectively. In the diabetes group, the participants’ average age was 58.6 years, with males accounting for 51.50% and whites representing 35.59%. In the prediabetes group, the participants’ average age was 52.0 years, with males accounting for 52.62% and whites representing 41.75%. For all-cause mortality, approximately one-third of the subjects died from cardiovascular disease both in the diabetes and prediabetes group. Therefore, we conducted an analysis of hazard ratios for both all-cause and CVD mortality. The subjects were categorized into three groups based on tertiles of NLR levels: tertile 1 (0.68–1.71), tertile 2 (0.71–2.48), and tertile 3 (2.48–7.58) for diabetes subjects and tertile 1 (0.62–1.60), tertile 2 (1.60–2.29), tertile 3 (2.29–6.23) for prediabetes subjects. For diabetes subjects, in comparison to the lower tertile of NLR, individuals in the upper tertile of NLR exhibited characteristics such as advanced age, male gender, non-Hispanic white ethnicity, higher educational attainment, and lower HbA1c levels. Additionally, they demonstrated a greater prevalence of alcohol consumption, smoking, hypertension, CVD, cancer, COPD, and anemia. Furthermore, this group displayed moderate family income, BMI, and eGFR. Such a trend was seen in prediabetes subjects, as indicated in Table 1.

**Table 1:** Baseline characteristics of diabetes and prediabetes subjects

Characteristic	Tertiles of NLR levels						Tertiles of NLR levels				
	Diabetes						Prediabetes				
	Total (*n* = 7246)	Tertile 1 (*n* = 2415)	Tertile 2 (*n* = 2406)	Tertile 3 (*n* = 2425)	*P* value		Total (*n* = 13,024)	Tertile 1 (*n* = 3911)	Tertile 2 (*n* = 3868)	Tertile 3 (*n* = 3887)	*P* value
NLR	2.34(0.02)	1.33(0.01)	2.08(0.01)	3.41(0.02)	< 0.0001		2.13(0.01)	1.26(0.01)	1.93(0.00)	3.11(0.01)	< 0.0001
Age, years	58.6( 0.26)	56.8(0.42)	58.1(0.42)	60.5(0.38)	< 0.0001		52.0(0.23)	50.6(0.35)	51.0(0.29)	54.3(0.31)	< 0.0001
Male	3732(51.50)	1113(44.64)	1223(50.88)	1396(56.72)	< 0.0001		6853(52.62)	2204(49.94)	2228(52.40)	2421(54.11)	0.02
Race/ethnicity					< 0.0001						< 0.0001
Hispanic Mexican	1604(22.14)	516(11.45)	580(10.61)	508( 8.52)			2355(18.08)	758(9.78)	873(9.84)	724(7.66)	
Non-Hispanic black	1699(23.45)	819(22.91)	501(12.18)	379( 8.06)			2744(21.07)	1412(19.18)	757( 9.34)	575( 6.72)	
Non-Hispanic white	2579(35.59)	585(47.63)	851(61.73)	1143(72.03)			5437(41.75)	1283(54.93)	1802(67.28)	2352(74.70)	
Others	1364(18.82)	495(18.00)	474(15.48)	395(11.39)			2488(19.1)	944(16.10)	847(13.54)	697(10.92)	
Education level					< 0.001						0.24
Less than high school	2768(38.2)	975(29.96)	921(24.90)	873(23.29)			3830(29.41)	1294(20.93)	1264(19.02)	1272(20.11)	
High school or equivalent	1654(22.83)	554(25.43)	504(23.43)	594(27.79)			3051(23.43)	999(23.45)	1030(24.94)	1022(25.68)	
College or above	2824(38.97)	886(44.61)	981(51.67)	958(48.93)			6143(47.17)	2104(55.62)	1985(56.04)	2054(54.21)	
Martial					0.71						0.01
Married/Cohabitating	4409(60.85)	1467(63.96)	1473(63.04)	1469(64.50)			8189(62.88)	2769(69.54)	2699(66.83)	2769(69.54)	
Not married	2837(39.15)	948(36.04)	933(36.96)	956(35.50)			4835(37.12)	1510(30.46)	1649(33.17)	1510(30.46)	
Family income-poverty ratio					< 0.001						0.03
<1	1675(23.12)	592(18.97)	544(15.47)	539(15.07)			2613(20.06)	916(14.95)	844(12.79)	853(13.36)	
1–3	3399(46.91)	1136(44.18)	1100(39.73)	1163(41.57)			5675(43.57)	1880(37.87)	1859(36.92)	1936(39.60)	
>=3	2172(29.98)	687(36.85)	762(44.79)	723(43.36)			4736(36.36)	1601(47.18)	1576(50.29)	1559(47.04)	
Drinking status					0.01						0.01
Non-drinker	1327(18.31)	486(18.40)	451(16.96)	390(13.60)			1893(14.53)	684(13.04)	612(10.41)	597(10.96)	
Ever drinker	5919(81.69)	1929(81.60)	1955(83.04)	2035(86.40)			11,131(85.47)	3713(86.96)	3667(89.59)	3751(89.04)	
Smoking status					0.004						< 0.0001
Never	3688(50.9)	1328(54.18)	1246(51.27)	1114(46.56)			6848(52.58)	2510(56.37)	2247(51.12)	2091(47.26)	
Ever	2392(33.01)	715(30.12)	783(31.80)	894(36.41)			3484(26.75)	1067(25.40)	1153(28.41)	1264(28.99)	
Current	1166(16.09)	372(15.70)	377(16.93)	417(17.04)			2692(20.67)	820(18.23)	879(20.47)	993(23.75)	
BMI, kg/m²	32.66( 0.14)	31.89(0.19)	33.04(0.22)	32.93(0.25)	< 0.0001		30.02(0.09)	29.56(0.13)	30.12(0.15)	30.33(0.16)	< 0.001
HbA1c,%	7.17(0.03)	7.26(0.05)	7.20(0.05)	7.08(0.05)	0.05		5.64(0.01)	5.66(0.01)	5.63(0.01)	5.62(0.01)	< 0.0001
History of CVD	1713(23.64)	449(16.90)	540(21.42)	724(26.71)	< 0.001		1442(11.07)	370( 7.92)	413( 8.40)	659(12.77)	< 0.0001
History of hypertension	5147(71.03)	1670(65.48)	1666(66.45)	1811(72.20)	< 0.001		6242(47.93)	1982(41.93)	1955(42.79)	2305(49.95)	< 0.0001
Hyperlipidemia	6260(86.39)	2075(86.90)	2082(88.40)	2103(87.73)	0.5		10,203(78.34)	3400(78.13)	3358(79.45)	3445(79.87)	0.31
History of cancer	937(12.93)	237(11.84)	312(14.61)	388(16.84)	0.003		1305(10.02)	340( 8.89)	378(10.10)	587(13.59)	< 0.0001
History of COPD	392(5.41)	86(3.43)	129(5.59)	177(7.04)	0.001		551(4.23)	137(3.77)	170(4.49)	244(5.29)	0.03
Depression status	1970(27.19)	637(24.44)	633(25.66)	700(26.52)	0.47		2538(19.49)	838(19.14)	801(18.49)	899(20.63)	0.16
Anemia	1081(14.92)	302( 9.43)	341(10.89)	438(13.25)	0.001		1054(8.09)	406(7.16)	285(4.54)	363(6.19)	< 0.0001
HEI scores	51.38(0.24)	52.08(0.41)	51.15(0.38)	51.03(0.38)	0.11		50.32(0.21)	50.93(0.30)	50.15(0.31)	49.94(0.32)	0.04
Physical activity					0.01						0.41
Low	3093(42.69)	1093(42.34)	1017(41.38)	983(36.93)			4537(34.84)	1521(34.62)	1496(35.33)	1520(33.65)	
High	4153(57.31)	1322(57.66)	1389(58.62)	1442(63.07)			8487(65.16)	2876(65.38)	2783(64.67)	2828(66.35)	

Data were presented as n (%) for categorical variables and mean (standard error) for continuous variables. All percentages and mean were adjusted for survey weights designed in NHANES. Tertiles of NLR in diabetes subjects: Tertile 1: 0.68–1.71; Tertlie 2: 1.71–2.48; Tertile 3: 2.48–7.58; Tertiles of NLR in prediabetes subjects: Tertile 1: 0.62–1.60; Tertile 2: 1.60–2.29; Tertile 3: 2.29–6.23. *P* < 0.05 means statistical significance. Abbreviate: *BMI* body mass index, *eGFR* estimated glomerular filtration rate, *HbA1c* glycated hemoglobin A1c, *CVD* cardiovascular disease, *COPD* chronic obstructive pulmonary disease, *HEI* healthy eating index

### All-cause and CVD mortality with tertiles of NLR levels and survival analyses

Over a period of 8.0 years, 1909 people with diabetes died, with 671 cases attributed to cardiovascular causes, 305 cases to cancer, and 933 cases to other causes. And over a period of 8.46 years, 1974 people with prediabetes died, with 616 cases attributed to cardiovascular causes, 479 cases to cancer, and 879 cases to other causes. The hazard ratios for all-cause and CVD mortality among individuals with diabetes and prediabetes, based on tertiles of NLR levels, are presented in Table 2a and Table 2b, respectively.

**Table 2a:** Hazard ratios for all-cause and CVD mortality by tertiles of NLR levels in subjects with diabetes

NLR	Per 1 unit increment	Tertiles of NLR levels			*P* for trend
		Tertile 1	Tertile 2	Tertile 3	
**All-cause mortality**					
No. deaths/total	1909/7246	494/2415	587/2406	828/2425	
Not adjusted	1.30(1.23, 1.37)	1.00(ref)	1.21(1.05, 1.40)	1.82(1.58, 2.09)	<0.0001
Model1	1.22(1.16, 1.28)	1.00(ref)	1.09(0.94, 1.27)	1.52(1.31, 1.77)	<0.0001
Model2	1.22(1.16, 1.28)	1.00(ref)	1.11(0.95, 1.29)	1.53(1.32, 1.78)	<0.0001
Model3	1.16(1.10, 1.23)	1.00(ref)	1.06(0.91, 1.23)	1.37(1.19, 1.58)	<0.0001
**CVD mortality**					
No. deaths/total	671/6008	163/2006	206/2003	302/1999	
Not adjusted	1.36(1.24, 1.50)	1.00(ref)	1.28(1.02, 1.61)	1.93(1.52, 2.45)	<0.0001
Model1	1.29(1.18, 1.41)	1.00(ref)	1.14(0.89, 1.45)	1.65(1.32, 2.08)	<0.0001
Model2	1.30(1.20, 1.42)	1.00(ref)	1.16(0.91, 1.48)	1.67(1.33, 2.10)	<0.0001
Model3	1.25(1.14, 1.37)	1.00(ref)	1.16(0.91, 1.48)	1.63(1.29, 2.05)	<0.0001

Model 1: Adjusted for age (< 65 or > = 65years), sex (male or female), race/ethnicity (Hispanic Mexican, non-Hispanic black, non-Hispanic white, or others), marital status (married/Cohabitating, not married), family income-to-poverty ratio (< 1.0, 1.0–3.0, or ≥ 3.0), education level (less than high school, high school or equivalent, or college or above);

Model 2: model 1 adjustments + BMI (< 30 or ≥ 30.0 kg/m2), smoking status (never, former, or current), drinking status (non-drinker or ever drinker), physical activity (low or high), HEI scores, cancer (no or yes);

Model 3: model 2 adjustments + eGFR (< 30, 30–60, >=60 ml/min/1.73 m²), HbA1c (< 7% or > = 7%), diabetes duration (< 10 or ≥ 10 years), anemia (no or yes), CVD (no or yes), hypertension (no or yes), hyperlipidemia (no or yes), depression (no or yes), COPD (no or yes), use of antidiabetic drug (no or yes), use of hypotensive drug (no or yes), use of lipid-lowering drug (no or yes)

**Table 2b:** Hazard ratios for all-cause and CVD mortality by tertiles of NLR levels in subjects with prediabetes

NLR	Per 1 unit increment	Tertiles of NLR levels			*P* for trend
		Tertile 1	Tertile 2	Tertile 3	
**All-cause mortality**					
No. deaths/total	1974/13,024	460/4397	584/4279	930/4348	
Not adjusted	1.40(1.33, 1.48)	1.00(ref)	1.17(0.97, 1.40)	1.83(1.59, 2.10)	<0.0001
Model1	1.20(1.13, 1.28)	1.00(ref)	1.09(0.91, 1.29)	1.37(1.18, 1.59)	<0.0001
Model2	1.18(1.11, 1.25)	1.00(ref)	1.06(0.90, 1.26)	1.33(1.14, 1.54)	<0.0001
Model3	1.12(1.05, 1.19)	1.00(ref)	1.05(0.88, 1.24)	1.21(1.03, 1.43)	0.01
**CVD mortality**					
No. deaths/total	616/11,666	121/3911	177/3868	318/3887	
Not adjusted	1.60(1.47, 1.75)	1.00(ref)	1.32(0.96, 1.80)	2.36(1.82, 3.06)	<0.0001
Model1	1.34(1.21, 1.48)	1.00(ref)	1.20(0.87, 1.64)	1.66(1.24, 2.21)	<0.0001
Model2	1.33(1.20, 1.48)	1.00(ref)	1.18(0.86, 1.61)	1.66(1.24, 2.22)	<0.0001
Model3	1.24(1.11, 1.37)	1.00(ref)	1.21(0.88, 1.67)	1.49(1.08, 2.04)	0.01

Model 1: Adjusted for age (< 65 or > = 65years), sex (male or female), race/ethnicity (Hispanic Mexican, non-Hispanic black, non-Hispanic white, or others), marital status (married/Cohabitating, not married), family income-to-poverty ratio (< 1.0, 1.0–3.0, or ≥ 3.0), education level (less than high school, high school or equivalent, or college or above);

Model 2: model 1 adjustments + BMI (< 30 or ≥ 30.0 kg/m2), smoking status (never, former, or current), drinking status (non-drinker or ever drinker), physical activity (low or high), HEI scores, cancer (no or yes);

Model 3: model 2 adjustments + eGFR (< 30, 30–60, >=60 ml/min/1.73 m²), anemia (no or yes), hypertension (no or yes), hyperlipidemia (no or yes), depression (no or yes), COPD (no or yes), use of hypotensive drug (no or yes), use of lipid-lowering drug (no or yes)

After adjusting for potential confounders, every unit increment in the absolute value of NLR, equivalent to 50SD in diabetes subjects or 100SD in prediabetes subjects, resulted in a 16% higher risk of mortality from any cause (HR, 1.16; 95% CI, 1.10–1.23) and a 25% higher risk of mortality from cardiovascular disease (HR 1.25; 95% CI 1.14–1.37) in diabetes subjects, and a 12% higher risk of mortality from overall cause (HR, 1.12; 95% CI, 1.05–1.19) and a 24% higher risk of mortality from cardiovascular disease (HR 1.24; 95% CI 1.11–1.37) in prediabetes subjects. The hazard ratios for all-cause mortality and CVD mortality after multiple adjustments in diabetes subjects, comparing high to low NLR tertile, were found to be 1.37 (95% CI, 1.19–1.58) and 1.63 (95% CI, 1.29–2.05), respectively. And the correlation between high to low NLR tertile and heightened susceptibility to mortality from any cause (HR, 1.21; 95% CI, 1.03–1.43) and CVD mortality (HR, 1.49; 95% CI, 1.08–2.04) remained statistically significant (both *p*-values for trend < 0.05) in prediabetes subjects.

**Fig. 2:**
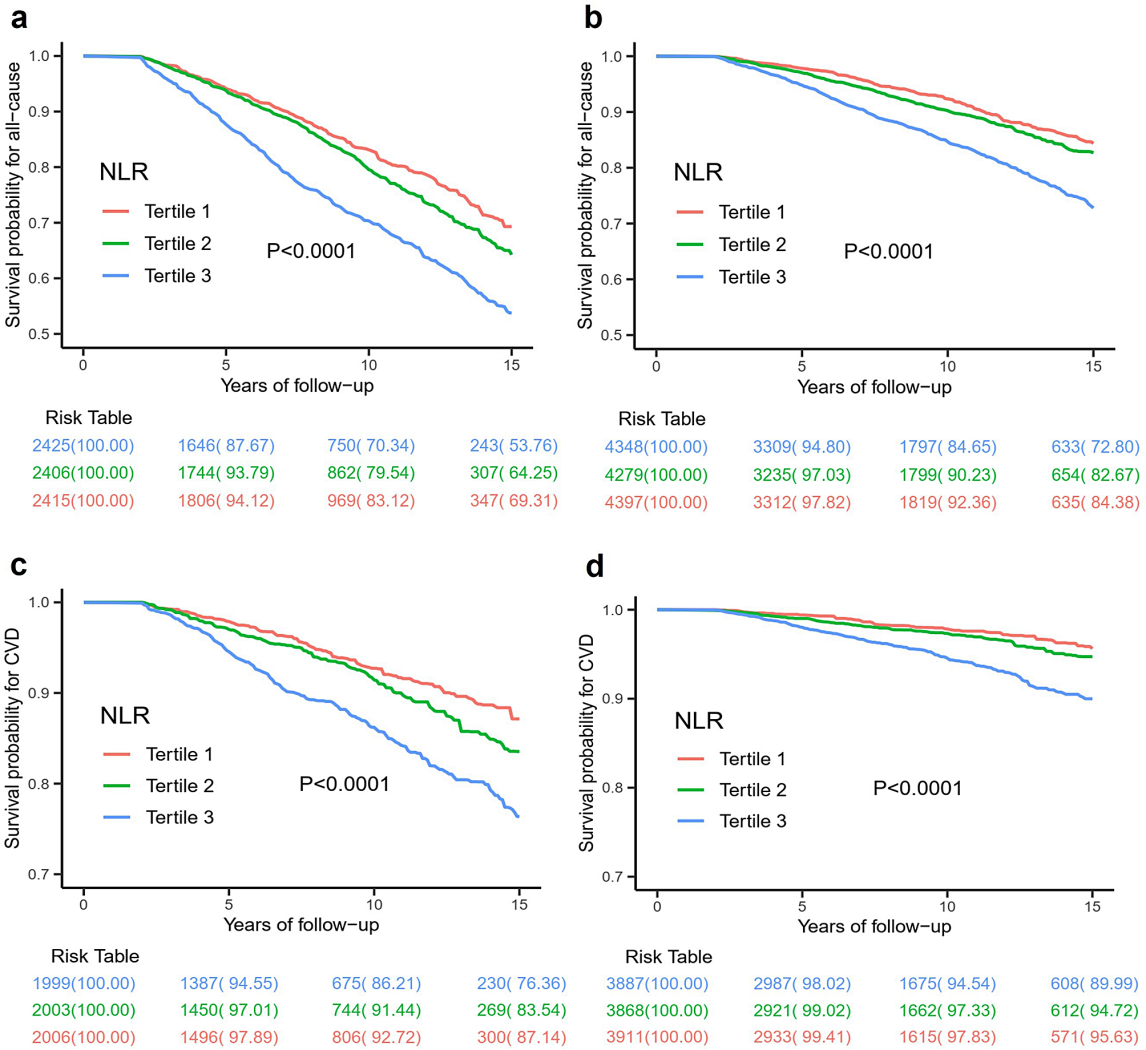
Weighted Kaplan–Meier survival curves for all-cause mortality for diabetes (**a**) and prediabetes (**b**) subjects according to tertiles of NLR. Tertiles of NLR in diabetes subjects: Tertile 1: 0.68–1.71; Tertile 2: 1.71–2.48; Tertile 3: 2.48–7.58; Tertiles of NLR in prediabetes subjects: Tertile 1: 0.62–1.60; Tertile 2: 1.60–2.29; Tertile 3: 2.29–6.23. Weighted Kaplan–Meier survival curves for CVD mortality for diabetes (**c**) and prediabetes (**d**) subjects according to tertiles of NLR. Tertiles of NLR in diabetes subjects: Tertile 1: 0.68–1.68; Tertlie 2: 1.68–2.42; Tertile 3: 2.42–7.58; Tertiles of NLR in prediabetes subjects: Tertile 1: 0.62–1.58; Tertile 2: 1.58–2.25; Tertile 3: 2.25–6.23

The Kaplan-Meier survival curves, adjusted for weights, were analyzed based on tertiles of NLR. The findings indicated that individuals in the highest tertile of NLR exhibited the lowest cumulative probability of survival for both all-cause and cardiovascular events, as depicted in Fig. 2. Specifically, when NLR levels exceeded 2.48 in diabetes participants, the 10-year cumulative survival probability was determined to be 70.34% for all-cause events and 86.21% for cardiovascular events. And when NLR levels exceeded 2.29 in prediabetes participants, the 10-year cumulative survival probability was found to be 84.65% for all-cause events and 94.54% for cardiovascular events.

**Fig. 3:**
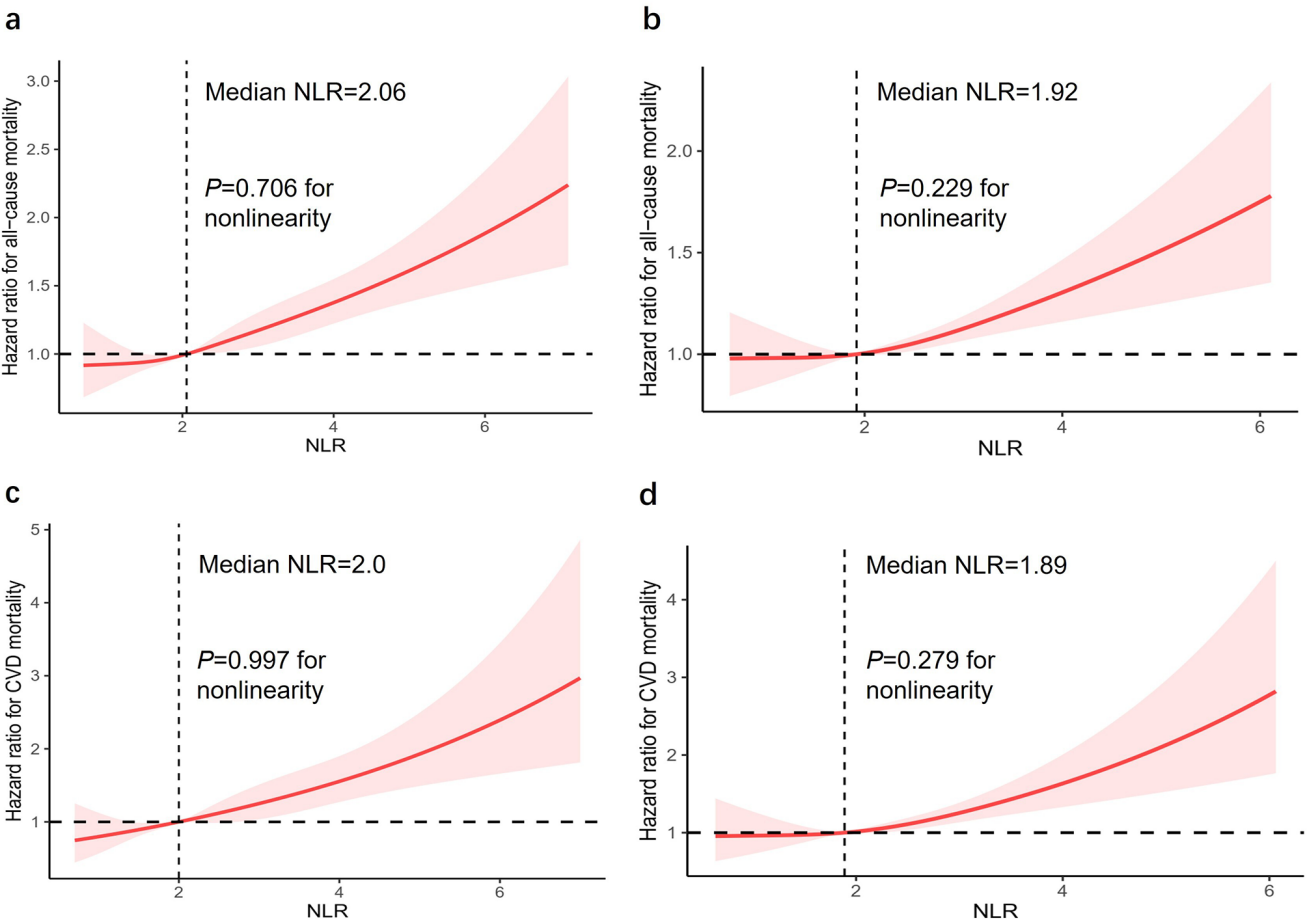
Dose-response associations between NLR and all-cause mortality in diabetes (**a**) and prediabetes (**b**) subjects. Both *p*-values for overall was 0. *p*-value for nonlinearity in diabetes and prediabetes subjects was 0.706 and 0.229, respectively. Dose-response associations between NLR and CVD mortality in diabetes (**c**) and prediabetes (**d**) subjects. Both p-values for overall was 0. *p*-value for nonlinearity in diabetes and prediabetes subjects was 0.997 and 0.279, respectively. The solid line and gray shading showed hazard ratios and 95% CIs, respectively. Models were adjusted for age, sex, race/ethnicity, education level, family income-poverty ratio, drinking status, smoking status, BMI, eGFR, CVD, hypertension, hyperlipidemia, cancer, COPD, depression status, anemia, physical activity, HEI scores, use of hypotensive drug, use of lipid-lowering drug. Diabetes duration, HbA1c, and use of antidiabetic drug were adjusted additionally for diabetes subjects

The RCS curves effectively depicted the non-linear correlation between NLR levels and both overall and cardiovascular mortality among adults with diabetes and prediabetes, following comprehensive adjustments as illustrated in Fig. 3. Significantly, there was a clear correlation between NLR levels and the mentioned mortality outcomes, demonstrating a dose-response relationship (*P*-value = 0). Furthermore, our analysis revealed a positive linear trend in the correlation between NLR levels and both overall and cardiovascular death, as evidenced by non-significant *P*-values for nonlinearity (*P*-value = 0.706 and 0.997, respectively) in diabetes group and nonlinearity (*P*-value = 0.229 and 0.279, respectively) in prediabetes group.

### Subgroup and sensitivity analyses

**Fig. 4:**
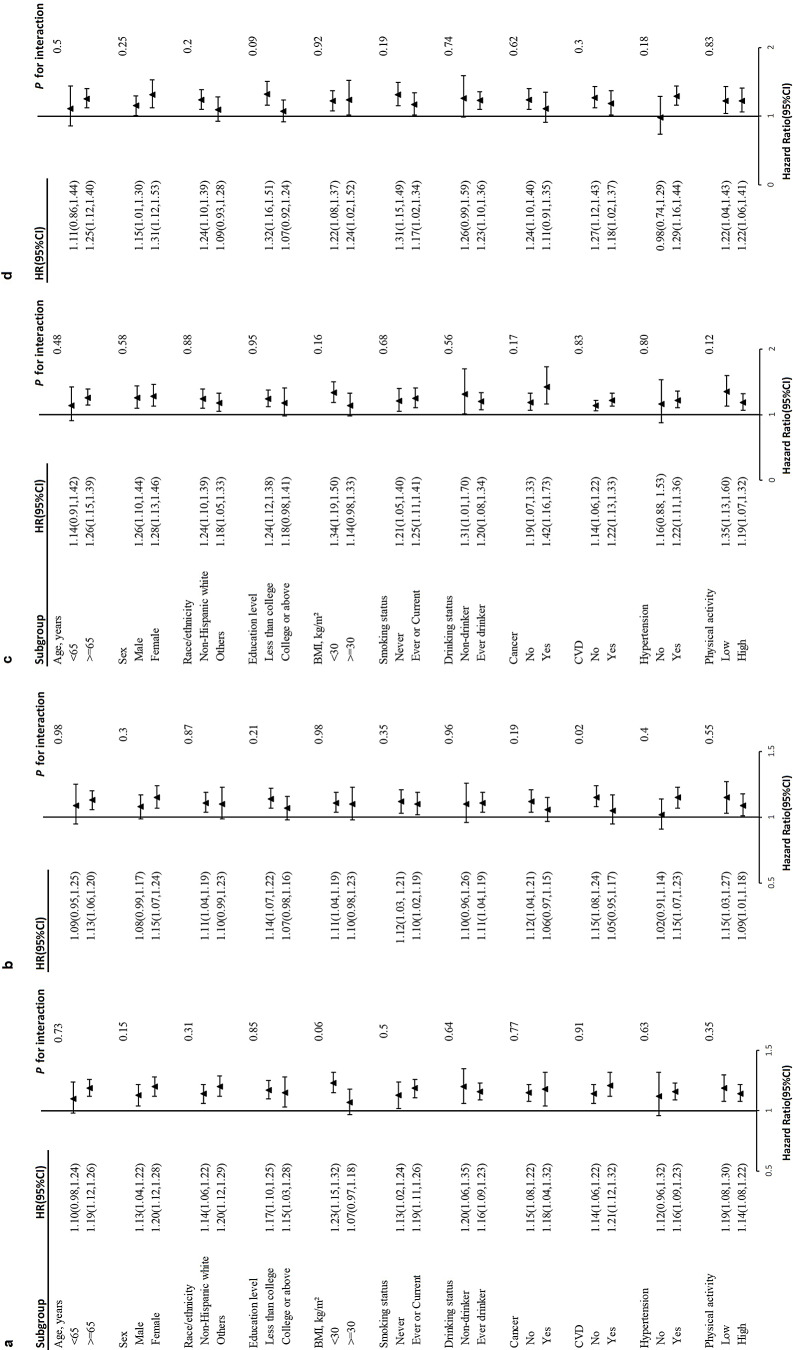
Stratified analyses of the association between NLR and all-cause mortality in diabetes (**a**) and prediabetes (**b**) subjects. Stratified analyses of the association between NLR and CVD mortality in diabetes (**c**) and prediabetes (**d**) subjects. Models were adjusted for age, sex, race/ethnicity, education level, family income-poverty ratio, drinking status, smoking status, BMI, eGFR, CVD, hypertension, hyperlipidemia, cancer, COPD, depression, anemia, physical activity, HEI scores, use of hypotensive drug, use of lipid-lowering drug except for the corresponding subgroup variables. Diabetes duration, HbA1c and use of antidiabetic drug were adjusted additionally for diabetes subjects

A noteworthy correlation was observed between NLR levels and baseline history of CVD in the prediabetes group for CVD mortality (*P* = 0.02 for interaction) (Fig. 4c, d). In the subset of prediabetes individuals without a prior history of CVD, the modified hazard ratio (95% CI) for CVD mortality was 1.15 (1.08, 1.24). On the other hand, in the subset of prediabetes individuals who have a previous CVD, the CVD mortality had an adjusted hazard ratio (95% CI) of 1.05 (0.95, 1.17). The association between NLR levels and overall mortality in hyperglycemia subjects remained stable irrespective of different stratifying factors (Fig. 4a, b), including age, gender, ethnicity, educational attainment, family income-poverty ratio, alcohol consumption, tobacco use, BMI, eGFR, CVD, hypertension, hyperlipidemia, cancer, COPD, depression, anemia, use of hypotensive drug, and use of lipid-lowering drug. Diabetes duration, HbA1c level, and use of antidiabetic drug were adjusted additionally for diabetes subjects.

In the subgroup without prior history of CVD, the multiple-adjusted hazard ratios for all-cause mortality and CVD mortality in diabetes subjects, comparing high to low NLR tertile, were found to be 1.33(95% CI, 1.09–1.61) and 1.93 (95% CI, 1.34–2.78), respectively. And the correlation between high to low NLR tertile and mortality from any cause (HR, 1.26; 95% CI, 1.04–1.54) remained statistically significant in prediabetes subjects (Table S1a and S1b). In the subgroup without a baseline history of CVD and cancer, the hazard ratios for overall and cardiovascular mortality exhibit similar significant differences (Table S3a and S3b). In the subgroup with a history of CVD, the hazard ratios for all-cause mortality after full adjustments in diabetes subjects, comparing high to low NLR tertile, were found to be 1.57 (95% CI, 1.28–1.593). And the correlation between high to low NLR tertile and mortality from CVD events (HR, 1.62; 95% CI, 1.05–2.50) remained statistically significant in prediabetes subjects (Table S2a and S2b).

## Discussion

Our study included 20,270 hyperglycemia subjects with a longer than 8 years of follow-up period. By the conclusion of the study, a total of 1909 deaths were recorded in the diabetes group, with 671 attributed to cardiovascular causes and 1238 attributed to other diseases. And a total of 1974 deaths were recorded in the prediabetes group, with 616 attributed to cardiovascular causes and 1358 attributed to other causes. The findings indicated that participants with elevated levels of NLR exhibited lower levels of HbA1c but a higher prevalence of cardiovascular disease and cancer at the beginning of the study. Additionally, these individuals demonstrated a higher incidence of both overall and cardiovascular mortality. Consequently, it can be inferred that heightened NLR levels may serve as an independent prognostic factor for mortality from any cause and cardiovascular disease in hyperglycemia people.

Many investigations have explored the correlation between NLR and the long-term complications and prognosis of diabetes [21, 22, 28]. For Scottish diabetic populations, higher NLR levels were found to increase the prevalence of retinopathy [21], particularly among individuals below the age of 65 and those with well-managed glycemic control. A study from Rio de Janeiro [29] noted that elevated NLR increased all-cause mortality by up to 19% in patients with type 2 diabetes mellitus (at 10.5 years of follow-up), but the sample size of the study was only 689 people. Through the analysis of a substantial sample size of 32,328 subjects from the NHANES database, Chen discovered a significant association between elevated NLR and increased risk of overall and cardiovascular death in the general population [30]. When the NLR value was greater than 3, the general population had a 43% increased risk of all-cause mortality and a 44% increased risk of cardiovascular mortality. In contrast, the NLR levels in our study were divided by tertiles, and when the NLR value was greater than 2.48, the diabetic population had a similar risk of all-cause mortality as the general population, but a higher risk of cardiovascular mortality. Such a trend was also significant in subjects with prediabetes. By analyzing seven cycles of 3251 diabetic patients in the NHANSE database, Dong et al. found a strong link between high NLR levels and increased risks of death and heart-related death in people with diabetes [31]. When the NLR was greater than 3.48, diabetic patients had a doubled risk of all-cause mortality and a 1.8-fold increase in cardiovascular mortality. However, this study failed to adequately incorporate covariates that affect the outcome, such as underlying cardiovascular disease, history of cancer, lifestyle, and medication use, and did not take into account subjects with prediabetes. While most studies have focused on the association of NLR with complications and poor prognosis of diabetes, few studies have been conducted on the association of NLR with poor prognosis of prediabetes. To our current understanding, our study represents the initial comprehensive examination of the relationship between NLR levels and mortality in both diabetes and prediabetes populations after full consideration of multivariable adjustments.

Our research found that diabetes individuals with NLR levels above 2.48 had a significantly higher risk of mortality from any cause (37%) and cardiovascular disease (63%) compared to those with lower NLR levels. Additionally, our dose-response analysis demonstrated a positive correlation between NLR levels and both all-cause and CVD mortality. After accounting for confounding factors in diabetes subjects, there was a 16% increased risk of all-cause mortality and a 25% increased risk of CVD mortality for every unit increase in the absolute value of NLR. The Kaplan-Meier survival plots indicated a notable correlation between heightened NLR levels and heightened susceptibility to mortality from any cause and cardiovascular disease. These findings provide compelling evidence for the close correlation between elevated NLR levels and unfavorable outcomes in terms of overall and cardiovascular mortality. In our study, prediabetes has a higher risk of death due to its progression to diabetes, highlighting the importance of inflammatory markers in predicting poor outcomes in those with high blood sugar. Therefore, it is plausible that NLR, serving as an inflammation marker, may possess intrinsic abilities to predict the probability of all-cause and cardiovascular mortality in individuals with diabetes and prediabetes. The evaluation of the NLR is subject to various influencing factors, including age, race, corticosteroid usage, and the presence of chronic diseases such as cancer, diabetes, obesity, depression, neoplasms, heart disease, and anemia. These factors impact the function, activity, behavior, and dynamic changes of neutrophil and lymphocyte counts [32–34]. Utilizing the extensive and reliable NHANES database, this study comprehensively accounted for confounding variables related to NLR. It ensured the credibility of NLR’s predictive value for mortality risk in hyperglycemia subjects. There is no normal reference range for NLR in healthy adults, and the average level of NLR is related to race [35–37]. Forget et al. [36] found that typical NLR values in a group of healthy, non-elderly people ranged from 0.78 to 3.53 in a sizable retrospective case-control study. In our research, we found a significant increase in deaths from any cause and cardiovascular events among diabetic adults with NLR levels above 2.48 and prediabetic adults with NLR levels above 2.29.

Our primary findings were robust and withstood rigorous sensitivity analyses. Without the influence of a baseline history of CVD and cancer, the relationship between different levels of NLR and mortality was still notable. In subgroup analyses, a noteworthy interaction is observed solely between NLR levels and baseline CVD history among prediabetes subjects. Within this subgroup, participants lacking a history of CVD exhibit a higher risk of overall mortality unexpectedly. This trend persists across three distinct models in sensitivity analyses, particularly among individuals with higher tertile NLR levels. The improvement can likely be credited to the common use of certain diabetes drugs, like GLP-1R agonists and SGLT-2 inhibitors, which have been shown to greatly enhance heart health and reduce death rates [38–40]. These drugs are often prescribed to diabetics with heart conditions, but people without known heart issues might not get them as promptly or at all. In diabetic participants with concurrent cardiovascular disease, the predictive value of NLR in determining cardiovascular mortality outcomes may be limited. In contrast, NLR may be more valuable in identifying adverse outcomes in participants who have not yet developed cardiovascular disease.

This prospective cohort study possesses several strengths. Notably, the routine administration of a peripheral whole blood test ensured the inclusion of a broader population, owing to its affordability and clinical relevance. As a result, the findings obtained in relation to the correlation between the NLR and the risk of mortality in individuals with diabetes and prediabetes are considered to be more reliable. Ultimately, 7246 eligible diabetes subjects and 13,024 eligible prediabetes subjects were successfully enrolled in the study. Afterward, considering that NLR is susceptible to a variety of possible factors and that there is a correlation between overall and cardiovascular mortality and hyperglycemia, we performed a thorough adjustment for known confounding variables. We developed several clinical models to fully assess the prognostic significance of NLR in the mortality of individuals with diabetes and prediabetes. Nonetheless, it is important to acknowledge that this study has some limitations, as certain unidentified confounding variables may not have been accounted for during the adjustment process. NHANES did not provide information on acute illnesses during blood collection or the reliability of neutrophil and lymphocyte counts. Participants with extreme NLR values, 1%, were removed from the analysis to reduce disease-related distortion and improve the accuracy of the initial NLR assessment. It is essential to highlight that NLR indicates the relative equilibrium between the body’s bone marrow and lymphocyte profiles, and monitoring the dynamic changes in NLR is crucial for predicting mortality outcomes in hyperglycemia adults. The NHANES database did not include any follow-up data on neutrophils and lymphocytes, thus precluding the ability to elucidate the influence of changes in NLR on mortality outcomes. Nevertheless, our study unequivocally demonstrates a positive association between elevated baseline NLR levels and increased risks of all-cause and cardiovascular mortality in participants with diabetes and prediabetes. Therefore, we expect that forthcoming prospective studies will be carried out to determine the exact significance of NLR in forecasting mortality associated with overall and cardiovascular mortality in adults with hyperglycemia.

## Conclusions

The measurement of NLR is readily accessible and economically viable in clinical practice. Our study has demonstrated the potential of NLR as a predictive factor for mortality in subjects with both diabetes and prediabetes. In addition to conventional risk factors, it is crucial to acknowledge the impact of low-grade inflammation on the adverse outcomes of all-cause and cardiovascular disease mortality in the hyperglycemia population. It’s crucial to explore other factors that could affect NLR and tackle the difficulties in tracking its changes over time.

### Electronic supplementary material

Below is the link to the electronic supplementary material.

**Supplementary Material 1: Table S1a** Hazard ratios of all-cause and CVD mortality by tertiles of NLR levels in diabetic subjects without baseline history of CVD. **Table S1b** Hazard ratios of all-cause and CVD mortality by tertiles of NLR levels in prediabetic subjects without baseline history of CVD. **Table S2a** Hazard ratios of all-cause and CVD mortality by tertiles of NLR levels in diabetic subjects with baseline history of CVD. **Table S2b** Hazard ratios of all-cause and CVD mortality by tertiles of NLR levels in diabetic subjects with baseline history of CVD. **Table S3a** Hazard ratios of all-cause and CVD mortality by tertiles of NLR levels in diabetic subjects without baseline history of CVD and cancer. **Table S3b** Hazard ratios of all-cause and CVD mortality by tertiles of NLR levels in prediabetic subjects without baseline history of CVD and cancer

## Data Availability

All data used and analyzed in this study were available on the NHANES website.

## Abbreviations

BMI: Body mass index
CAPI: Computer-Assisted Personal Interviewing
CIs: Confidence intervals
COPD: Chronic obstructive pulmonary disease
CVD: Cardiovascular disease
eGFR: Estimated glomerular filtration rate
HbA1c: Glycated hemoglobin A1c
HDL-C: High-density lipoprotein cholesterol
HEI: Healthy eating index
HRs: Hazard ratios
LDL-C: Low-density lipoprotein cholesterol
NHANES: National Health and Nutrition Examination Survey
NLR: Neutrophil-lymphocyte ratio
TC: Total cholesterol
TG: Triglyceride
US: United States
