# Supplementary material for: Association of neutrophil-lymphocyte ratio with all-cause and cardiovascular mortality in US adults with diabetes and prediabetes: a prospective cohort study
Source: BMC Endocr Disord. 2024 May 10;24:64. doi: 10.1186/s12902-024-01592-7 (PMC11084136; doi:10.1186/s12902-024-01592-7)
Supplement: Supplementary file 1 — Supplementary Material 1: Table S1a Hazard ratios of all-cause and CVD mortality by tertiles of NLR levels in diabetic subjects without baseline history of CVD. Table S1b Hazard ratios of all-cause and CVD mortality by tertiles of NLR levels in prediabetic subjects without baseline history of CVD. Table S2a Hazard ratios of all-cause and CVD mortality by tertiles of NLR levels in diabetic subjects with baseline history of CVD. Table S2b Hazard ratios of all-cause and CVD mortality by tertiles of NLR levels in diabetic subjects with baseline history of CVD. Table S3a Hazard ratios of all-cause and CVD mortality by tertiles of NLR levels in diabetic subjects without baseline history of CVD and cancer. Table S3b Hazard ratios of all-cause and CVD mortality by tertiles of NLR levels in prediabetic subjects without baseline history of CVD and cancer [file 12902_2024_1592_MOESM1_ESM.docx]

**Table S1a**Hazard ratios of all-cause and CVD mortality by tertiles of NLR levels in diabetic subjects without baseline history of CVD

| **NLR** | **Per 1 unit** | **Tertiles of NLR levels** | | | ***P* for trend** |
| --- | --- | --- | --- | --- | --- |
|  | **increment** | **Tertile 1** | **Tertile 2** | **Tertile 3** |  |
| **All-cause mortality** |  |  |  |  |  |
| No. deaths/total | 1143/5533 | 314/1872 | 341/1815 | 488/1846 |  |
| Not adjusted | 1.24(1.15, 1.34) | 1.00(ref) | 1.15(0.96, 1.37) | 1.61(1.33, 1.94) | ＜0.0001 |
| Model1 | 1.18(1.10, 1.27) | 1.00(ref) | 1.10(0.92, 1.31) | 1.42(1.17, 1.73) | ＜0.001 |
| Model2 | 1.18(1.10, 1.27) | 1.00(ref) | 1.13(0.94, 1.36) | 1.42(1.17, 1.73) | ＜0.001 |
| Model3 | 1.15(1.06, 1.23) | 1.00(ref) | 1.13(0.94, 1.34) | 1.33(1.09, 1.61) | 0.005 |
| **CVD mortality** |  |  |  |  |  |
| No. deaths/total | 359/4749 | 92/1592 | 101/1574 | 166/1583 |  |
| Not adjusted | 1.44(1.25, 1.66) | 1.00(ref) | 1.30(0.94, 1.79) | 2.17(1.53, 3.07) | ＜0.0001 |
| Model1 | 1.40(1.23, 1.59) | 1.00(ref) | 1.20(0.86, 1.67) | 2.03(1.43, 2.90) | ＜0.0001 |
| Model2 | 1.39(1.23, 1.58) | 1.00(ref) | 1.23(0.87, 1.73) | 2.05(1.42, 2.95) | ＜0.0001 |
| Model3 | 1.35(1.18, 1.55) | 1.00(ref) | 1.19(0.84, 1.69) | 1.93(1.34, 2.78) | ＜0.001 |

Model 1: Adjusted for age (<65 or >=65years), sex (male or female), race/ethnicity (Hispanic Mexican, non-Hispanic black, non-Hispanic white, or others), marital status (married/Cohabitating, not married), family income-to-poverty ratio (< 1.0, 1.0–3.0, or ≥3.0), education level (less than high school, high school or equivalent, or college or above);

Model 2: model 1 adjustments + BMI (< 30 or ≥30.0 kg/m2), smoking status (never, former, or current), drinking status (non-drinker or ever drinker), physical activity (low or high), HEI scores, cancer (no or yes);

Model 3: model 2 adjustments + eGFR (<30, 30-60, >=60ml/min/1.73m²), HbA1c (<7% or >=7%), diabetes duration (< 10 or ≥10 years), anemia (no or yes), hypertension (no or yes), hyperlipidemia (no or yes), depression (no or yes), COPD (no or yes), use of antidiabetic drug (no or yes), use of hypotensive drug (no or yes), use of lipid-lowering drug (no or yes)

**Table S1b**Hazard ratios of all-cause and CVD mortality by tertiles of NLR levels in prediabetic subjects without baseline history of CVD

| **NLR** | **Per 1 unit** | **Tertiles of NLR levels** | | | ***P* for trend** |
| --- | --- | --- | --- | --- | --- |
|  | **increment** | **Tertile 1** | **Tertile 2** | **Tertile 3** |  |
| **All-cause mortality** |  |  |  |  |  |
| No. deaths/total | 1419/11582 | 340/3887 | 420/3848 | 659/3847 |  |
| Not adjusted | 1.37(1.28, 1.47) | 1.00(ref) | 1.12(0.90, 1.40) | 1.74(1.46, 2.07) | ＜0.0001 |
| Model1 | 1.23(1.14, 1.32) | 1.00(ref) | 1.06(0.86, 1.30) | 1.40(1.16, 1.69) | ＜0.0001 |
| Model2 | 1.20(1.12, 1.29) | 1.00(ref) | 1.04(0.85, 1.28) | 1.36(1.12, 1.64) | ＜0.001 |
| Model3 | 1.16(1.08, 1.24) | 1.00(ref) | 1.00(0.81, 1.23) | 1.26(1.04, 1.54) | 0.01 |
| **CVD mortality** |  |  |  |  |  |
| No. deaths/total | 388/10551 | 87/3517 | 114/3517 | 187/3517 |  |
| Not adjusted | 1.52(1.35, 1.71) | 1.00(ref) | 1.08(0.73, 1.58) | 1.90(1.38, 2.61) | ＜0.0001 |
| Model1 | 1.34(1.17, 1.53) | 1.00(ref) | 0.98(0.68, 1.43) | 1.44( 1.02, 2.02) | 0.02 |
| Model2 | 1.34(1.18, 1.53) | 1.00(ref) | 0.98(0.68, 1.40) | 1.46( 1.04, 2.06) | 0.01 |
| Model3 | 1.29(1.14, 1.45) | 1.00(ref) | 0.91(0.62, 1.33) | 1.37(0.97, 1.94) | 0.03 |

Model 1: Adjusted for age (<65 or >=65years), sex (male or female), race/ethnicity (Hispanic Mexican, non-Hispanic black, non-Hispanic white, or others), marital status (married/Cohabitating, not married), family income-to-poverty ratio (< 1.0, 1.0–3.0, or ≥3.0), education level (less than high school, high school or equivalent, or college or above);

Model 2: model 1 adjustments + BMI (< 30 or ≥30.0 kg/m2), smoking status (never, former, or current), drinking status (non-drinker or ever drinker), physical activity (low or high), HEI scores, cancer (no or yes);

Model 3: model 2 adjustments + eGFR (<30, 30-60, >=60ml/min/1.73m²), anemia (no or yes), hypertension (no or yes), hyperlipidemia (no or yes), depression (no or yes), COPD (no or yes), use of hypotensive drug (no or yes), use of lipid-lowering drug (no or yes)

**Table S2a**Hazard ratios of all-cause and CVD mortality by tertiles of NLR levels in diabetic subjects with baseline history of CVD

| **NLR** | **Per 1 unit** | **Tertiles of NLR levels** | | | ***P* for trend** |
| --- | --- | --- | --- | --- | --- |
|  | **increment** | **Tertile 1** | **Tertile 2** | **Tertile 3** |  |
| **All-cause mortality** |  |  |  |  |  |
| No. deaths/total | 766/1713 | 221/575 | 240/567 | 305/571 |  |
| Not adjusted | 1.30(1.21, 1.39) | 1.00(ref) | 1.00(0.80, 1.23) | 1.76(1.45, 2.13) | ＜0.0001 |
| Model1 | 1.25(1.16, 1.35) | 1.00(ref) | 0.90(0.73, 1.12) | 1.61(1.31, 1.98) | ＜0.0001 |
| Model2 | 1.27(1.18, 1.38) | 1.00(ref) | 0.90(0.72, 1.13) | 1.67(1.35, 2.07) | ＜0.0001 |
| Model3 | 1.23(1.13, 1.33) | 1.00(ref) | 0.90(0.72, 1.12) | 1.57(1.28, 1.93) | ＜0.0001 |
| **CVD mortality** |  |  |  |  |  |
| No. deaths/total | 312/1259 | 89/372 | 101/369 | 122/374 |  |
| Not adjusted | 1.20(1.07, 1.36) | 1.00(ref) | 0.87(0.63, 1.21) | 1.37(0.96, 1.95) | 0.08 |
| Model1 | 1.15(1.01, 1.30) | 1.00(ref) | 0.79(0.55, 1.12) | 1.30(0.90, 1.88) | 0.13 |
| Model2 | 1.18(1.04, 1.33) | 1.00(ref) | 0.78(0.54, 1.12) | 1.35(0.92, 2.00) | 0.09 |
| Model3 | 1.12(1.00, 1.26) | 1.00(ref) | 0.85(0.59, 1.24) | 1.34(0.92, 1.94) | 0.10 |

Model 1: Adjusted for age (<65 or >=65years), sex (male or female), race/ethnicity (Hispanic Mexican, non-Hispanic black, non-Hispanic white, or others), marital status (married/Cohabitating, not married), family income-to-poverty ratio (< 1.0, 1.0–3.0, or ≥3.0), education level (less than high school, high school or equivalent, or college or above);

Model 2: model 1 adjustments + BMI (< 30 or ≥30.0 kg/m2), smoking status (never, former, or current), drinking status (non-drinker or ever drinker), physical activity (low or high), HEI scores, cancer (no or yes);

Model 3: model 2 adjustments + eGFR (<30, 30-60, >=60ml/min/1.73m²), HbA1c (<7% or >=7%), diabetes duration (< 10 or ≥10 years), anemia (no or yes), hypertension (no or yes), hyperlipidemia (no or yes), depression (no or yes), COPD (no or yes), use of antidiabetic drug (no or yes), use of hypotensive drug (no or yes), use of lipid-lowering drug (no or yes)

**Table S2b**Hazard ratios of all-cause and CVD mortality by tertiles of NLR levels in prediabetic subjects with baseline history of CVD

| **NLR** | **Per 1 unit** | **Tertiles of NLR levels** | | | ***P* for trend** |
| --- | --- | --- | --- | --- | --- |
|  | **increment** | **Tertile 1** | **Tertile 2** | **Tertile 3** |  |
| **All-cause mortality** |  |  |  |  |  |
| No. deaths/total | 555/1442 | 151/481 | 174/480 | 230/481 |  |
| Not adjusted | 1.25(1.14, 1.38) | 1.00(ref) | 1.07(0.85, 1.36) | 1.63(1.29, 2.06) | ＜0.0001 |
| Model1 | 1.08(0.97, 1.19) | 1.00(ref) | 0.97(0.76, 1.23) | 1.12(0.86, 1.45) | 0.35 |
| Model2 | 1.06(0.96, 1.18) | 1.00(ref) | 0.96(0.75, 1.22) | 1.08(0.84, 1.40) | 0.49 |
| Model3 | 1.05(0.95, 1.16) | 1.00(ref) | 0.98(0.77, 1.25) | 1.04(0.79, 1.37) | 0.76 |
| **CVD mortality** |  |  |  |  |  |
| No. deaths/total | 228/1115 | 44/372 | 74/369 | 110/374 |  |
| Not adjusted | 1.45(1.24, 1.69) | 1.00(ref) | 1.64(1.05, 2.57) | 2.81(1.78, 4.41) | ＜0.0001 |
| Model1 | 1.20(1.03, 1.41) | 1.00(ref) | 1.46(0.94, 2.26) | 1.79(1.16, 2.77) | 0.01 |
| Model2 | 1.19(1.02, 1.39) | 1.00(ref) | 1.44(0.93, 2.22) | 1.71(1.12, 2.62) | 0.01 |
| Model3 | 1.17(1.01, 1.36) | 1.00(ref) | 1.57(1.01, 2.45) | 1.62(1.05, 2.50) | 0.04 |

Model 1: Adjusted for age (<65 or >=65years), sex (male or female), race/ethnicity (Hispanic Mexican, non-Hispanic black, non-Hispanic white, or others), marital status (married/Cohabitating, not married), family income-to-poverty ratio (< 1.0, 1.0–3.0, or ≥3.0), education level (less than high school, high school or equivalent, or college or above);

Model 2: model 1 adjustments + BMI (< 30 or ≥30.0 kg/m2), smoking status (never, former, or current), drinking status (non-drinker or ever drinker), physical activity (low or high), HEI scores, cancer (no or yes);

Model 3: model 2 adjustments + eGFR (<30, 30-60, >=60ml/min/1.73m²), anemia (no or yes), hypertension (no or yes), hyperlipidemia (no or yes), depression (no or yes), COPD (no or yes), use of hypotensive drug (no or yes), use of lipid-lowering drug (no or yes)

**Table S3a**Hazard ratios of all-cause and CVD mortality by tertiles of NLR levels in diabetic subjects without baseline history of CVD and cancer

|  | **Per 1 unit** | **Tertiles of NLR levels** | | | ***P* for trend** |
| --- | --- | --- | --- | --- | --- |
|  | **increment** | **Tertile 1** | **Tertile 2** | **Tertile 3** |  |
| **All-cause mortality** |  |  |  |  |  |
| No. deaths/total | 949/4922 | 262/1651 | 283/1638 | 404/1633 |  |
| Not adjusted | 1.24(1.14, 1.35) | 1.00(ref) | 1.14(0.93, 1.41) | 1.61(1.31, 1.96) | ＜0.0001 |
| Model1 | 1.18(1.10, 1.27) | 1.00(ref) | 1.11(0.91, 1.34) | 1.44(1.16, 1.79) | ＜0.001 |
| Model2 | 1.18(1.10, 1.26) | 1.00(ref) | 1.11(0.91, 1.35) | 1.42(1.14, 1.76) | 0.002 |
| Model3 | 1.13(1.04, 1.22) | 1.00(ref) | 1.09(0.89, 1.32) | 1.30(1.05, 1.62) | 0.01 |
| **CVD mortality** |  |  |  |  |  |
| No. deaths/total | 313/4286 | 82/1432 | 89/1409 | 142/1445 |  |
| Not adjusted | 1.38(1.18, 1.62) | 1.00(ref) | 1.20(0.85, 1.68) | 1.91(1.34, 2.74) | ＜0.001 |
| Model1 | 1.35(1.17, 1.55) | 1.00(ref) | 1.17(0.83, 1.65) | 1.80(1.24, 2.63) | 0.002 |
| Model2 | 1.35(1.17, 1.55) | 1.00(ref) | 1.15(0.81, 1.64) | 1.76(1.19, 2.60) | 0.004 |
| Model3 | 1.29(1.11, 1.49) | 1.00(ref) | 1.09(0.76, 1.56) | 1.66(1.12, 2.45) | 0.01 |

Model 1: Adjusted for age (<65 or >=65years), sex (male or female), race/ethnicity (Hispanic Mexican, non-Hispanic black, non-Hispanic white, or others), marital status (married/Cohabitating, not married), family income-to-poverty ratio (< 1.0, 1.0–3.0, or ≥3.0), education level (less than high school, high school or equivalent, or college or above);

Model 2: model 1 adjustments + BMI (< 30 or ≥30.0 kg/m2), smoking status (never, former, or current), drinking status (non-drinker or ever drinker), physical activity (low or high), HEI scores, cancer (no or yes);

Model 3: model 2 adjustments + eGFR (<30, 30-60, >=60ml/min/1.73m²), HbA1c (<7% or >=7%), diabetes duration (< 10 or ≥10 years), anemia (no or yes), hypertension (no or yes), hyperlipidemia (no or yes), depression (no or yes), COPD (no or yes), use of antidiabetic drug (no or yes), use of hypotensive drug (no or yes), use of lipid-lowering drug (no or yes)

**Table S3b**Hazard ratios of all-cause and CVD mortality by tertiles of NLR levels in prediabetic subjects without baseline history of CVD and cancer

|  | **Per 1 unit** | **Tertiles of NLR levels** | | | ***P* for trend** |
| --- | --- | --- | --- | --- | --- |
|  | **increment** | **Tertile 1** | **Tertile 2** | **Tertile 3** |  |
| **All-cause mortality** |  |  |  |  |  |
| No. deaths/total | 1135/10563 | 275/3520 | 342/3505 | 518/3538 |  |
| Not adjusted | 1.37(1.27, 1.48) | 1.00(ref) | 1.14(0.87, 1.49) | 1.71(1.41, 2.07) | ＜0.0001 |
| Model1 | 1.25(1.15, 1.35) | 1.00(ref) | 1.06(0.81, 1.39) | 1.40(1.14, 1.71) | ＜0.001 |
| Model2 | 1.23(1.13, 1.33) | 1.00(ref) | 1.05(0.81, 1.37) | 1.35(1.10, 1.66) | 0.001 |
| Model3 | 1.18(1.08, 1.28) | 1.00(ref) | 1.00(0.77, 1.30) | 1.26(1.02, 1.56) | 0.01 |
| **CVD mortality** |  |  |  |  |  |
| No. deaths/total | 324/9752 | 72/3252 | 95/3272 | 157/3228 |  |
| Not adjusted | 1.52(1.34, 1.72) | 1.00(ref) | 1.07(0.72, 1.59) | 1.98(1.44, 2.70) | ＜0.0001 |
| Model1 | 1.39(1.21, 1.59) | 1.00(ref) | 1.00( 0.67, 1.47) | 1.64(1.18, 2.27) | ＜0.001 |
| Model2 | 1.40(1.22, 1.60) | 1.00(ref) | 0.98( 0.67, 1.44) | 1.66(1.20, 2.30) | ＜0.001 |
| Model3 | 1.35(1.18, 1.53) | 1.00(ref) | 0.92(0.63, 1.36) | 1.57(1.13, 2.19) | 0.002 |

Model 1: Adjusted for age (<65 or >=65years), sex (male or female), race/ethnicity (Hispanic Mexican, non-Hispanic black, non-Hispanic white, or others), marital status (married/Cohabitating, not married), family income-to-poverty ratio (< 1.0, 1.0–3.0, or ≥3.0), education level (less than high school, high school or equivalent, or college or above);

Model 2: model 1 adjustments + BMI (< 30 or ≥30.0 kg/m2), smoking status (never, former, or current), drinking status (non-drinker or ever drinker), physical activity (low or high), HEI scores;

Model 3: model 2 adjustments + eGFR (<30, 30-60, >=60ml/min/1.73m²), anemia (no or yes), hypertension (no or yes), hyperlipidemia (no or yes), depression (no or yes), COPD (no or yes), use of hypotensive drug (no or yes), use of lipid-lowering drug (no or yes)
